# Digital Competence of Rural Teachers in Depopulated Regions of Spain: A Bibliometric Review

**DOI:** 10.3390/ejihpe15010005

**Published:** 2025-01-07

**Authors:** Pablo Fernández-Arias, María Sánchez-Jiménez, Álvaro Antón-Sancho, María Nieto-Sobrino, Diego Vergara

**Affiliations:** Technology, Instruction and Design in Engineering and Education Research Group (TiDEE.rg), Catholic University of Ávila, C/Canteros s/n, 05005 Ávila, Spain; pablo.fernandezarias@ucavila.es (P.F.-A.); maria.sanchezjimenez@ucavila.es (M.S.-J.); alvaro.anton@ucavila.es (Á.A.-S.); maria.nieto@ucavila.es (M.N.-S.)

**Keywords:** depopulation, teachers, digital competence, local development, bibliometric review, rural schools

## Abstract

Rural teachers have the potential to be important agents of local development. To achieve this goal, they need to acquire high digital competence in order to effectively integrate technology into their pedagogical practices, thus enriching the learning experience of students and fostering their participation. Digital competence contributes to reducing the education gap between urban and rural areas, promoting educational equity and inclusion. High digital competence also enables rural teachers to address the specific challenges of their environment, such as cultural diversity, scarce resources, and low population density. Against this backdrop, this article presents a bibliometric review of the importance of digital competence in rural teachers in Spain, where the problem of rural depopulation, as in other regions of Europe, has been accentuated in recent years. The objective of the bibliometric review is both (i) to find the strengths and weaknesses that concern researchers in relation to the digital training of teachers in rural areas and (ii) to express them explicitly in order to contribute to propose solutions. The results reveal the growing academic and political attention being paid to this issue, highlighting the need for rural teachers to acquire digital skills to adapt to current educational demands. In addition, they point to the importance of developing specific policies and programs in Europe as well as providing training opportunities and ongoing support to ensure that teachers in rural contexts can acquire or strengthen their digital competence, thereby improving the quality of education in these areas.

## 1. Introduction

**Figure 1:**
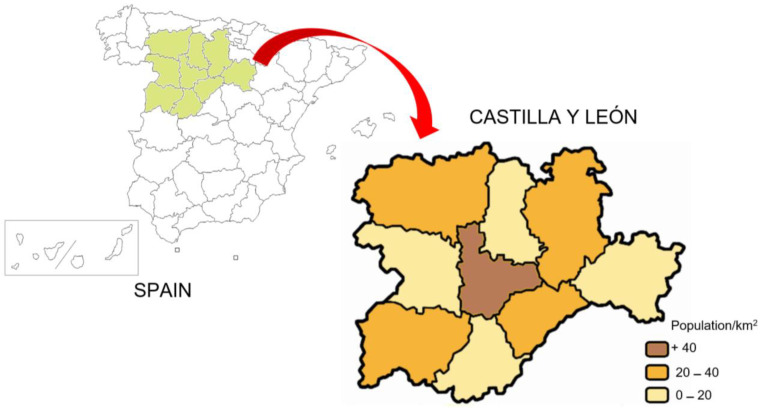
Population density (population/km^2^) in the different provinces of Castilla y León.

The high level of depopulation in rural areas in Spain has triggered the well-known term “Empty Spain”, which has become a serious social problem. Regions such as Castilla y León, Galicia, and Asturias have experienced a significant loss of population in their rural areas in recent years (Llorent-Bedmar et al., 2021). This phenomenon has had numerous consequences: (i) lack of opportunities in the rural environment (Lichter & McLaughlin, 1995); (ii) abandonment of numerous municipalities (Hoggart & Paniagua, 2001); (iii) high economic depression (Giménez & Montero, 2015); (iv) impoverished cultural life (Signes-Pont et al., 2022); and (v) closure of numerous rural schools (Castelló, 2023). In addition, depopulation is one of the main problems in rural areas in decline, due to the socioeconomic deterioration that pushes a greater number of young people towards the decision to emigrate (García-Arias et al., 2021). In regions suffering from depopulation, the rural exodus exceeds the number of births, which can reduce population density to critical situations (Escudero et al., 2019). A typical example of the emptying of Spain is the region of Castilla y León, which, with an area of over 90,000 km^2^, has a population of 2.4 million inhabitants, a figure that implies an average population density of close to 20 inhabitants per km^2^ (Junta de Castilla y León, 2024). This depopulation process has dragged on for more than half a century; since 1996, the region has been losing about 5000 inhabitants per year (Fernández-Arias et al., 2023a). As shown in Figure 1, some of the provinces most affected by depopulation in the Castilla y León region are Ávila, Soria, Palencia, and Zamora, with a population density of less than 20 hab/km^2^ (Ministerio de Agricultura, Pesca y Alimentación, 2024).

This phenomenon of rural depopulation is not exclusive to Castilla y León; many other regions of southern Europe, such as certain rural areas of Portugal, Italy, and Greece, also suffer from it (Pinilla et al., 2008). The centralization of services and opportunities in large cities has exacerbated this situation (Papadopoulos & Baltas, 2024). In northern Europe, countries such as Finland and Sweden are also facing problems related to rural depopulation, which are also influenced by climatic factors that make life in remote rural areas even more difficult (Makkonen & Inkinen, 2023; Pettersson, 2001).

This depopulation of rural areas has led to the closure of many rural schools, thus further complicating the social and economic situation of these territories (Barakat, 2014). The closure of the rural school marks the demographic death of the area in which it is located, because there will be no young parents left in the area, let alone new parents moving in (Barakat, 2014). Therefore, the Grouped Rural Center (GRC), the name given to grouped rural schools in Spain, represents an important solution to the educational challenges posed by the depopulation of these areas (Conde, 2006). These centers bring together several rural schools in several villages under the same administrative structure and make it possible to offer early childhood education in localities that otherwise would not have access to these basic services due to low population density and a declining birth rate (Polgár & Duguleana, 2015).

Rural schools are located in population centers in which agricultural activities predominate, which contrasts with the more endowed and sought-after city schools (Díaz, 2000). These centers offer an educational model that is adapted to the peculiarities of the rural environment, promoting cooperation and intergenerational learning, and in turn, they encourage repopulation by attracting families seeking a more personalized education and a different lifestyle (Santamaría-Cárdaba & Gallego, 2020). Access to the education system has improved significantly in recent years due to digital development (Li et al., 2021). That said, there are still large differences between urban and rural geographical areas—both in the quality of infrastructures and educational practices, where rural areas show lower levels of availability—in the adoption and usage of new educational technologies (Guillén-Gámez & Mayorga-Fernández, 2022). These disadvantages are also present among teachers working in these rural areas, due to the lack of digital resources (Guillén-Gámez et al., 2023) and the absence of teacher training resulting in an increased digital divide (Guillén-Gámez & Mayorga-Fernández, 2022). Therefore, traditional teaching, especially in rural environments, needs to be integrated with new forms of teaching and communication through the adoption of original methodologies and activities and the use of digital educational resources, web applications, and even e-learning platforms (Stenman & Pettersson, 2020).

In the GRCs and in the rural areas in which they are located, the rural teacher plays a crucial role, often being the main link between education and the community (White, 2015). The teacher is the most important factor in the teaching–learning process because his or her main function is to organize content and activities to be congruent with the needs of the students, thus practicing their ability to think, manage, and apply knowledge in daily life (Díaz, 2000; Kanokorn et al., 2014; Thompson et al., 2013) In addition, the teacher must have a high degree of digital competence so that he/she can make the most of educational technology to improve his/her teaching and learning processes (Guillén-Gámez & Mayorga-Fernández, 2022). The rural teacher, as a relevant actor in the environment, is obliged to develop, in addition to his or her specific competence, other types of competencies related to (i) digitalization, (ii) soft skills, and (iii) the promotion of local development (Alonso et al., 2023). The current context is marked by a constantly evolving information- and knowledge-based society in which soft and digital skills have emerged as a fundamental pillar in the field of education (Tsekeris, 2019). Technological advances around the world and the accelerated development of information technology are also present in education and science (Zhang, 2012). Many of these global changes occurred during the pandemic and had a direct impact on the educational system (Dekimpe et al., 2000) and require a different approach to education (Khoa & Huynh, 2024).

Rural teachers, as mediators of learning, face the challenge of adapting to these changes to respond to the needs of a generation of digital natives through digital competence (National Center for Education Statistics, 2023; Pagani et al., 2016). Digital competence is defined as the ability to explore and adapt flexibly in situations involving new technologies, allowing the analysis, selection and critical evaluation of data and information (Freiman et al., 2017). It is also defined as the set of knowledge, skills and abilities that enable the teacher to solve pedagogical problems using digital technology (Rubio-Gragera et al., 2023). This competence goes beyond the simple use of technologies in the teaching and learning process; it also implies a reflection on how these tools transform educational experiences. Its main objective is to maximize the opportunities offered by technologies to enrich and optimize educational practice, thus promoting a more dynamic and interactive approach in the learning environment (García-Delgado et al., 2023). In addition, teachers need to be trained and periodically improve their knowledge and skills to maintain a high level of quality in the performance of their professional role in the teaching–learning process (Ribosa et al., 2024). This situation contrasts significantly with the technological progress of society. Therefore, it is necessary to focus on enhancing the digital competencies of students in training, approaching them from a holistic perspective that focuses on instrumental, pedagogical and personal ambitions, with the aim of promoting a more inclusive and effective digital citizenship (Gabarda-Méndez et al., 2023). Therefore, teachers must have a minimum level of digital competence to correctly use technologies and methodologies in the teaching of specific contents, thereby adapting to their educational context and available resources (García-Vandewalle et al., 2023).

Nowadays, not knowing the potential of and how to use technology seriously limits an individual’s capacity for development and learning. The digital competence of the teacher indirectly influences the skills of the students, since the teacher is the reference and the example to be followed by younger generations (Chrásková & Chráska, 2021).

**Figure 2:**
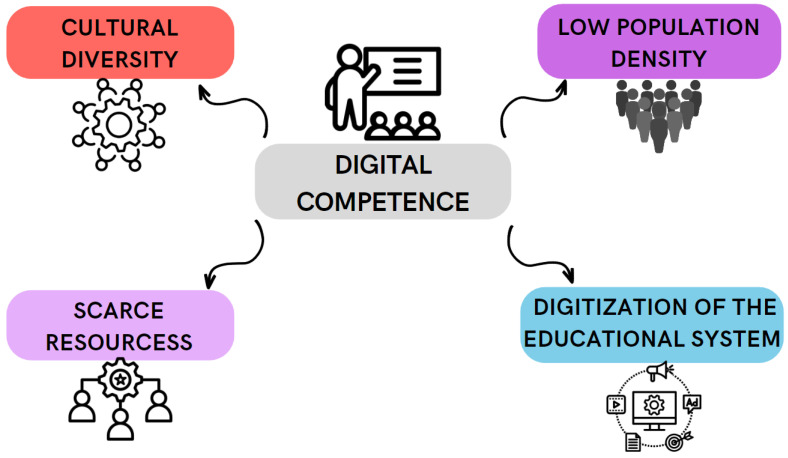
Digital competences of teachers in rural schools.

Therefore, high digital competence enables rural teachers to face great challenges: (i) the cultural diversity of their environment; (ii) the scarce economic and technical resources at their disposal; (iii) the low population density around them; and (iv) dealing with the digitization of the educational system effectively (Figure 2). Global online educational resources and communication tools facilitate the integration of diverse cultural perspectives and international collaboration, thus enriching learning and enhancing teaching skills (Gisbert-Cervera & Caena, 2022).

In view of this scenario, the purpose of the present research is to conduct a bibliometric analysis of the research developed in Spain on digital competence in teachers in rural areas. Consequently, the research objectives are as follows. RQ1: to identify the current level of digital competence in teachers in depopulated rural regions of Spain; and RQ2: to analyze the main trend lines in terms of research on teachers’ digital competence in depopulated rural areas of Spain.

It should be noted that the findings obtained in the present research in the context of Spain can be extrapolated to other regions of Europe that present a similar socioeconomic environment and also suffer from the problem of drastic depopulation in their rural environment (Pinilla et al., 2008). To achieve its research objectives, this study presents a bibliometric review of papers related to the digital competence of rural school teachers.

## 2. Materials and Methods

**Figure 3:**
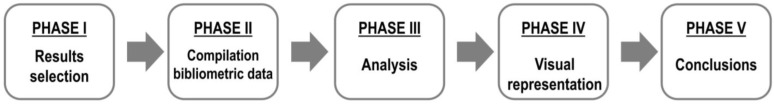
Investigative phases.

The bibliometric approach employed in this study encompasses several distinct phases (Figure 3): Phase I: The selection of relevant results is carried out, which is crucial in establishing a solid basis for the study. During this phase, specific inclusion and exclusion criteria are defined. This exhaustive search identifies relevant publications that meet the established standards of quality and relevance. The filtering process ensures that only the most significant studies are considered, which guarantees the validity of subsequent analyses. Phase II focuses on the gathering and organization of bibliometric data. In this stage, key data such as authors, years of publication, number of citations, and institutional affiliations are extracted. These data are meticulously organized into a structured database that facilitates subsequent analysis. Phase III entails a comprehensive analysis of the collected data. This analysis includes examination of temporal trends, identification of influential authors and institutions, and assessment of patterns of international collaboration. In addition, keyword co-occurrence analysis is used to identify emerging themes and novel areas of research. The advanced statistical techniques applied in this phase allow for the discovery of hidden patterns and significant relationships within the dataset, providing an in-depth understanding of the field under study. Phase IV is dedicated to the visual representation of the findings. Visual representation is a powerful tool for communicating complex findings in a clear and accessible way. Finally, Phase V concludes with the synthesis of insights and the drawing of final conclusions. The main conclusions and their implications for the specific field are identified, contextualizing the findings within the broader framework of the existing literature.

In relation to the selection of results (Phase I, Figure 3), it was decided to use the Scopus bibliographic database to cover as many scientific results as possible. Scopus is one of the most important and widely used bibliographic databases and offers results of high scientific quality (Fernández-Arias et al., 2023b). The collection of articles was carried out in May 2024. According to other examples of bibliometric studies (Vergara et al., 2024), to guarantee an exhaustive search and guarantee the identification of the most relevant articles on the topic, several keywords combined with Boolean operators should be used (Table 1).

**Figure 4:**
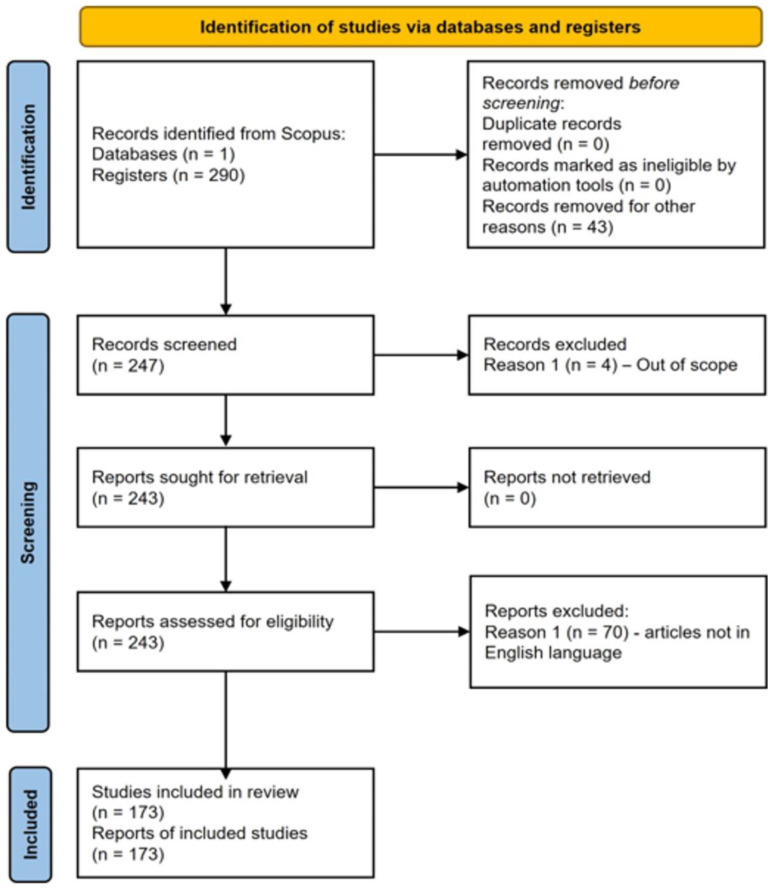
PRISMA 2020 checklist developed in this bibliometric review.

The preliminary keyword search revealed a total of 290 published results. To enhance the accuracy of the sample size determination, the PRISMA 2020 checklist (Abelha et al., 2020; David et al., 2023) was followed as a framework (Figure 4). In the screening phase, four of the results obtained were excluded because they were outside the scope of the research. The exclusion criteria defined were that the articles should be published in scientific journals and that they should be written in English. By focusing on articles in English, we facilitated access to high-impact research and ensured greater uniformity in the terminology used. Having defined these exclusion criteria, 70 results were excluded and 173 were considered for further analysis. The full list of selected articles is available to the scientific community at the end of the article. In conducting the bibliometric analysis and examining the data, the Bibliometrix R-package and VOSviewer^®^ were utilized. The definition of exclusion criteria for the results obtained, as well as the use of several standardized software tools and the participation of different researchers in the selection phase of the studies included, reduced the risk of bias in the subsequent bibliometric analysis.

## 3. Results

**Figure 5:**
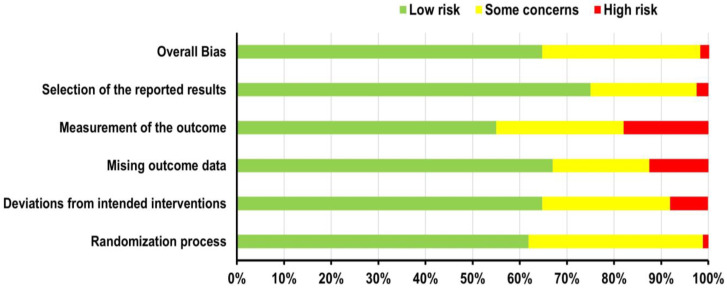
Analysis of risk of bias.

Using the RoB2 tool (Lucena-Anton et al., 2022), the overall bias assessment (Alfaqeeh et al., 2023; Mahmoud et al., 2024) for the 173 included papers (Figure 5) revealed that 55% (95 studies) were identified as having a low risk of bias across all five domains. Additionally, 20.5% (35 studies) showed ’some concerns’ in at least one domain, while 1.2% (2 studies) were classified as having a high risk of bias in at least one of the five domains. In terms of overall bias, 64.7% (112 results) were classified as low-risk, while 33.5% (58 results) were classified with ‘some concerns’, and only 2% (3 results) were considered high-risk. Therefore, it is possible to state that the data collection, statistical analysis, and interpretation of the results were carried out properly, with no significant systematic errors that could have affected the objectivity or validity of the conclusions. The likelihood that the results of this study are influenced by factors that could distort its conclusions is minimal.

The results obtained in Phase III (Figure 3) are shown in this section. Table 2 presents the primary data from the conducted bibliometric analysis. The initial study found in Scopus on the digital competencies of rural teachers dates from 2007 (Cordon et al., 2007). This article was developed at the University of Granada (Spain) and analyzes a procedure used to create a specific service structure to manage the promotion of the use of both e-learning and ICT in the teaching–learning process; however, it did not clearly address the issue of digital competencies of teachers in rural areas. Since 2007, the annual growth rate in research on the digital competencies of rural teachers has been 11.64%.

**Figure 6:**
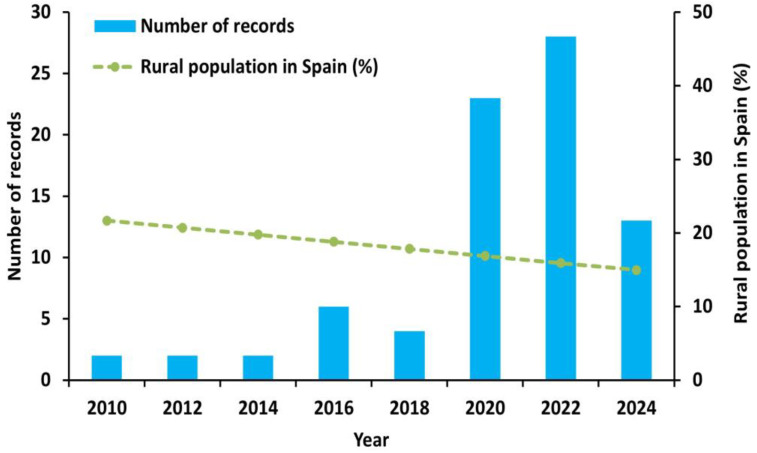
Variation in the number of articles on the digital competence of rural school teachers since 2010.

According to the first research question (RQ1), the evolution of results on the digital competence of teachers in rural schools from 2010 to 2024 is shown in Figure 6. From 2020 onwards, 23 articles were found, which reflects a substantial increase in the volume of research articles. However, the rural population in Spain is close to 8 million inhabitants, with a rate of decrease of 7% between 2011 and 2020, which means an annual fall in the rural population of close to 50,000 inhabitants and that, in general terms, the rural population in Spain is around 15% (Ministerio de Agricultura, Pesca y Alimentación, 2024). Given this situation, the scientific community has begun to increase its interest in the digital competence of teachers in rural areas of Spain. This phenomenon of rural depopulation has raised not only demographic but also social and educational concerns (Pinilla et al., 2008). Faced with this situation, the scientific community has begun to increase its interest in the digital competence of teachers in rural areas of Spain.

**Figure 7:**
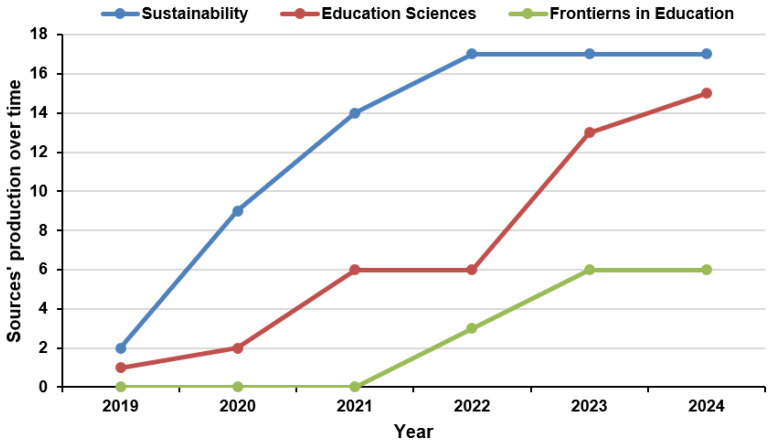
Sources’ production over time (2019–2024).

On the other hand, among the 78 identified journals, the most impactful one regarding the digital competence of teachers in rural schools is “Sustainability (Switzerland)” (H-index of 10), in which about 10% (17 absolute records) are published. Another journal that also has great results is “Education Sciences” (H-index of 6), in which about 6% are published. The third in the ranking is “Frontiers in Education” (H-index of 3,) in which about 3% are published. The remaining journals identified exert a residual influence of less than 3% within the published results. Figure 7 shows that the production of sources has grown over time. Since 2019, this growth has begun to accelerate, and publications in these journals have increased. “Sustainability (Switzerland)” has a predominant position, and “Education Sciences” has experienced an upward trend since 2022.

The local impact of the five main authors during 2007–2024 is shown in Table 3. The three most important authors are Cabero-Almenara, J. (h-index 9, 13 results); Palacios-Rodríguez, A. (h-index 9, 14 results), and Guillén-Gámez, F.D. (h-index 8, 13 results).

In the productivity of the authors, which is evaluated by the number of articles published, Cabero-Almenara J. stands out as the most prolific author. The authors with the most citations per year are Cabero-Almenara J., Palacios Rodríguez A., and Guillén-Gámez F.D. In accordance with Lotka’s law (Hinojo-Lucena et al., 2019), 504 of the authors identified (91% of the total) have only published one article. Furthermore, there are only three authors who have written more than 10 articles on the digital competence of teachers in rural Spain.

**Figure 8:**
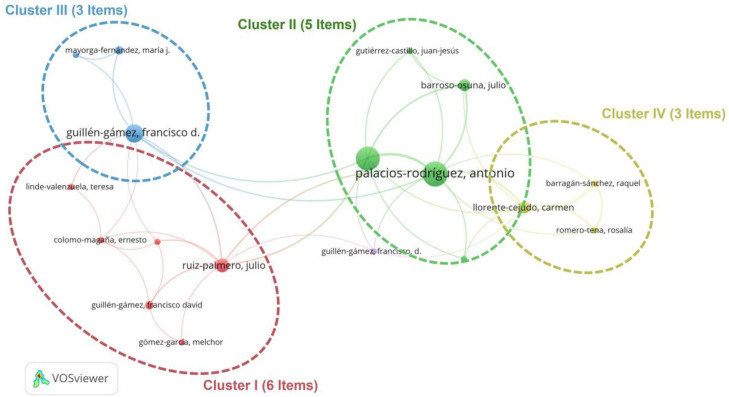
Co-citation network analysis (2007–2024). Source: own elaboration using VOS Viewer software 1.6.16.

Figure 8 illustrates the results of the authors’ analysis in terms of citation network. Co-citation analysis identifies which authors are most representative based on the frequency of their co-citations by other researchers (Fernández-Arias et al., 2023b). The results show that there are four most significant groups or sets of authors in terms of the digital skills of teachers in rural Spain. In Cluster I (network), there are six authors with minor relevance, while in Cluster II (green), there are five authors, among which Palacios and Barroso-Osuna stand out. Finally, in Cluster III (blue) and IV (yellow), there are three authors, including Guillén-Gámez. It is necessary to highlight the central role, in terms of co-citation, played by the authors Palacios-Rodríguez, Ruiz-Palomero, Guillén-Gámez, and Llorente-Cejudo.

Table 4 provides a comprehensive analysis of the five most influential countries in research on digital skills among teachers in rural areas of Spain. This analysis encompasses the number of published articles, single-country publications (SCPs), multi-country collaborative publications (MCPs), and the MCP/TP ratio, where TP denotes the total number of publications (MCP-Ratio).

If the analysis of research on the digital competence of teachers in rural Spain (RQ1) is carried out in terms of the most relevant affiliations (Table 5), as already observed in the top five countries (Table 4), the first country in terms of number of articles is Spain, and the University of Seville tops the list of most relevant affiliations, followed by the University of Granada. The University of Burgos and the University of Salamanca are the last affiliations in this top five.

Table 6 shows the countries with the highest number of citations in research on the digital competence of teachers in rural Spain. Spain, the United Kingdom, the USA, Norway, Sweden, China, and Australia are the leading countries in terms of citations. Notably, the USA ranks among the countries with the highest number of citations, despite not being one of the most productive countries, as shown in Table 3.

Going deeper into the citation analysis, upon examining the articles with the most citations (Table 7), the article “Generation Z’s teachers and their digital skills” (Fernández-Cruz & Fernández, 2016) has the highest number of overall citations, i.e., 214 citations and an average of 23.7 citations per year, followed by “Beyond COVID-19 supernova. Is another education coming?” (Azorín, 2020), with 157 citations and an average of 31.4 citations per year.

**Figure 9:**
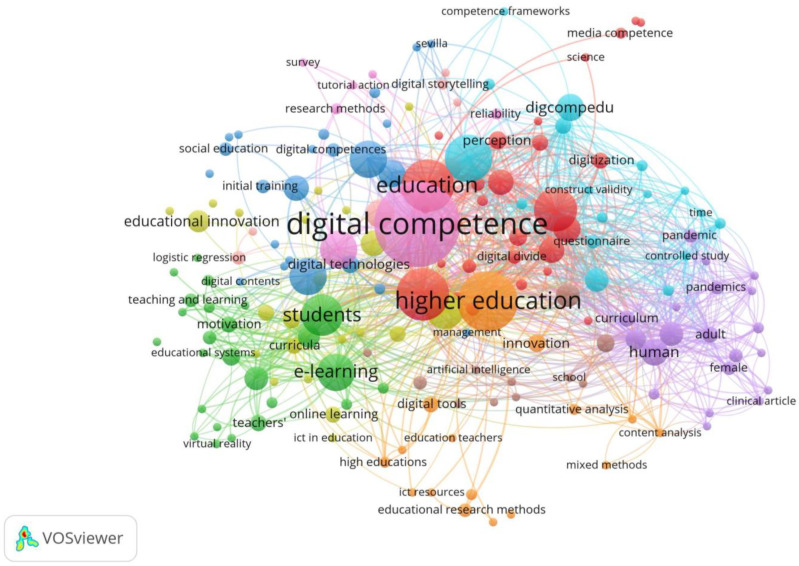
The main keyword structure (2007–2024).

Keywords play a crucial role in the indexing process and in enabling search engines to find and retrieve pertinent articles efficiently. They help in categorizing and locating relevant research by matching specific terms with the content of the articles. In this way, database users will be able to find the keywords provided by the authors, which will increase the number of readers of the work and, consequently, the citations received (Tejedor et al., 2020). In addition, keywords were used to identify research trends in the digital competencies in teachers in rural Spain (RQ2). In the keyword analysis, through concurrence (Figure 9), of the 173 results found, 784 keywords were identified. Of these, 29% of the total number of keywords appeared more than five times, while 35.46% of the total were repeated more than twice. It can be seen that the most relevant keywords are digital competence, education, students, and e-learning.

The keyword “digital competence” stands out above the rest, underscoring its centrality in current academic discourse. This finding indicates a significant focus on the development of digital competence as an essential component of digital competence research. In addition, related terms such as “teaching” and “higher education” suggest that much of the research focuses on how these competencies are integrated into teaching and learning processes, especially in rural faculties in depopulated regions of Spain.

Likewise, the relevant presence of the keyword “COVID-19” highlights how this health crisis has accelerated the adoption of digital technologies by rural teachers in hollowed-out areas of Spain. This context has led to an increased focus on modalities such as “e-learning”, evidencing a shift towards online learning platforms. In addition, terms such as “teacher training” and “ICT” highlight the importance of training teachers to effectively use digital technologies in their pedagogical practices. Considering the high clustering of keywords in the central part of the network, it is possible to affirm that there is a close relationship between all of them.

## 4. Discussion

**Figure 10:**
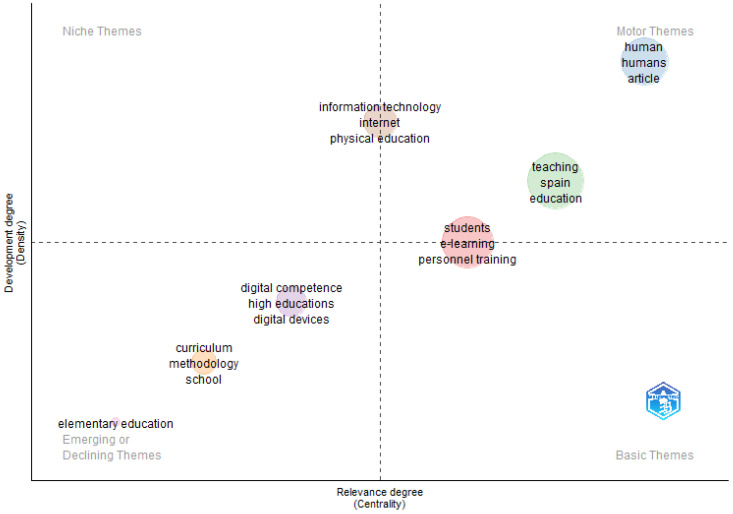
Thematic map (2007–2024).

To demonstrate the level of advancement and significance of ongoing research while highlighting emerging research trends, a thematic map has been constructed (Figure 10). This map features bubbles that denote clusters of keywords, with each bubble representing a specific research topic. The size and placement of these bubbles are based on the frequency and co-occurrence of the keywords, reflecting their prominence and relevance in the field: (i) niche themes (high development and low relevance), in which there are no reference themes; (ii) motor themes (high development and relevance), among which those related to “human” stand out; (iii) emerging/declining themes (low density and centrality), among which are “curriculum”, “digital competence” and “digital devices”; and (iv) basic themes (low development and high relevance), in which there are no reference themes. It is worth noting that “students”, “e-learning” and “personnel training” are among the motor and basic theme quadrants, which reflects the importance of these topics.

**Figure 11:**
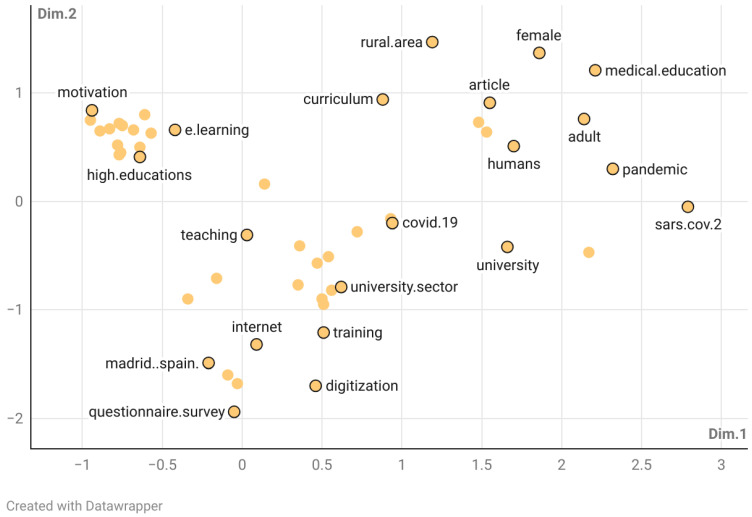
Multiple correspondence analysis (MCA; 2007–2024).

Figure 11 illustrates the multiple correspondence analysis (MCA) of the keywords, providing an in-depth view of the proximity and divergence within the research domain. This spatial distribution helps to highlight the focal areas and emerging trends within the research landscape, providing insights into the evolving priorities and interests within the field. This map enhances our ability to identify relationships between various variables with greater precision (Baiyegunhi et al., 2022; Iman et al., 2023).

The conceptual structure maps generated from this analysis reveal two primary dimensions (Dim. 1 and Dim. 2) derived from the factor analysis of the dataset. These dimensions offer a more nuanced understanding of the underlying structure and interconnections among the research variables (Nica & Chiriță, 2024). The terms located closer to the center of the map, such as motivation, e-learning, and learning systems, have garnered significant attention in recent years, indicating their prominence in current research. Conversely, terms that are more dispersed towards the periphery of the map correspond to research topics that have been less frequently discussed (Ejaz et al., 2022).

In Dim. 1, there is a clear differentiation between terms with high positive values, such as “adult” (2.14), “sars.cov.2” (2.79) and “pandemics” (2.32), which suggest a focus on adult education and medical contexts, possibly influenced by the COVID-19 pandemic. On the other hand, terms with negative values, such as “motivation” (−0.94) and “curricula” (−0.89), indicate an emphasis on more specific aspects of educational practice and research, suggesting a diversity of approaches within the educational field.

In Dim. 2, traditional educational approaches are contrasted with technological innovations. Highly negative terms, such as “questionnaire survey” (−1.94) and “digitization” (−1.70), reflect research methods and concepts related to the digital transition in education. In contrast, positive terms such as “e-learning” (0.66) and “educational technology” (0.80) indicate a strong focus on the use of educational technologies and digital resources. In addition, there is a significant clustering of terms related to teacher training, such as “teacher training” and “teachers,” underscoring the importance of preparing educators to meet the challenges of the digital age. Taken together, these results show how various aspects such as technology, teacher training, and geographical context are interrelated in the field of study of the digital competence of rural teachers in depopulated regions of Spain.

**Figure 12:**
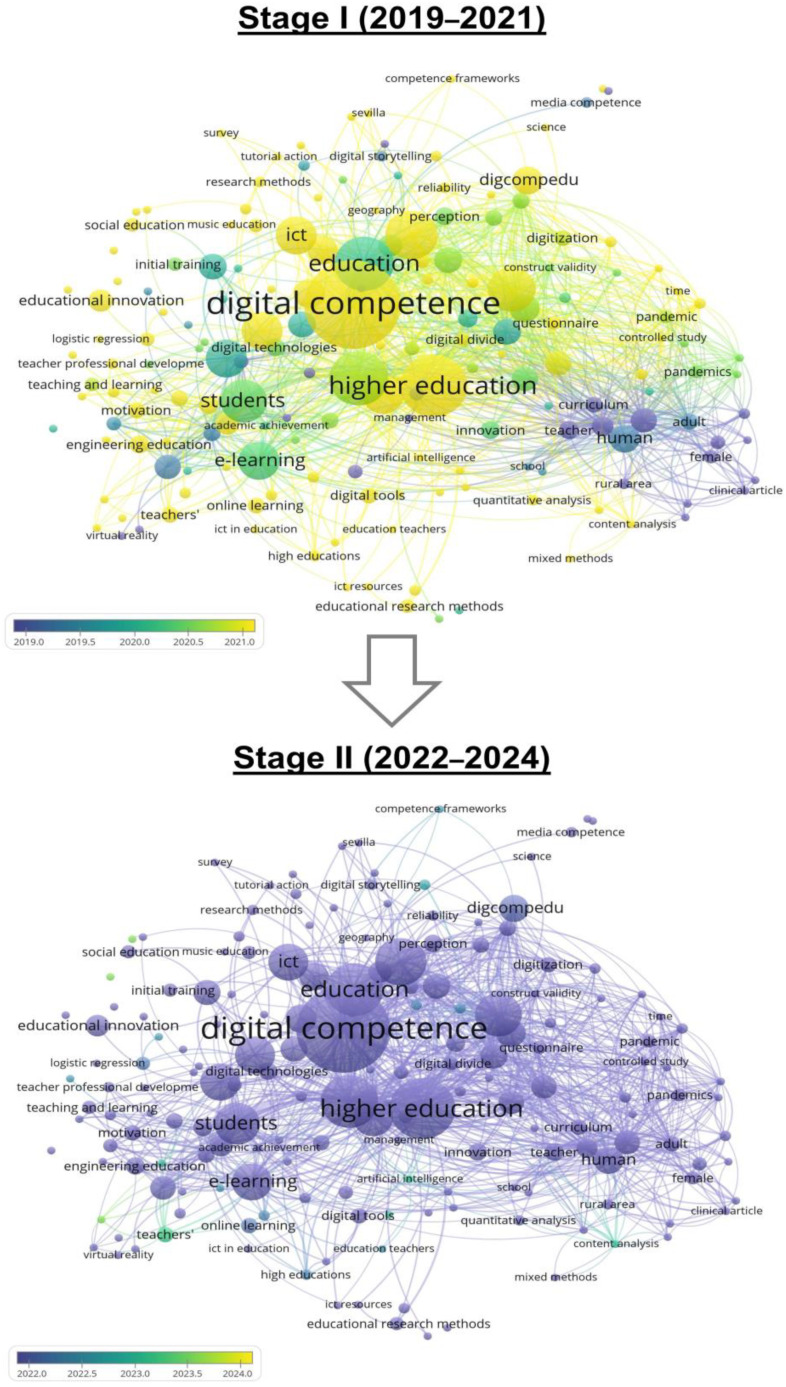
Keyword trends (2019–2021 and 2022–2024). Source: own elaboration using VOS Viewer software 1.6.16.

If the keyword analysis is performed according to time, it is observed that the most relevant terms occur from 2019 to 2022, a period that coincides with the highest productivity (Figure 6). If two time periods are analyzed, that is, 2019–2021 (Figure 12, Stage I) and 2022-2024 (Figure 12, Stage II), it is possible to observe that in Stage I, the most representative keywords were “digital competence”, “higher education”, “education” or “students”, and that the most used keywords throughout 2019 were human, computers or pandemic. However, in the period 2022–2024, it is observed that most of the keywords have had a greater influence throughout the year 2022.

Following these analyses, it is possible to observe the scarcity of issues related to the digital competence and the capacity of rural teachers to foster local development in their area of influence. Despite the growing awareness of the crucial role that educators and other local actors can play in the revitalization of rural communities in some regions of Spain (Barke & Newton, 1997), there are no relevant issues within the scientific community. This lack of comprehensive research on the capacity of rural teachers to foster local development limits the understanding of the dynamics between rural education and community development but also impedes the formulation of more effective educational policies and practices that could enhance the positive impact of teachers in their communities. It is difficult to make a reliable comparison of the digital skills of teachers in depopulated areas of different countries because there are a wide variety of social, demographic, and economic variables that can have an influence (Guillén-Gámez et al., 2023). Moreover, even differences between the digital competencies of teachers in urban and rural schools (RQ1) can be mentioned (Lin et al., 2023). The integration of STEAM degrees in modern education fosters creativity and critical thinking, skills that are enhanced by teachers with high digital competence (Sánchez-Jiménez et al., 2024). However, it would be interesting to focus on the case of Latin America, because it is a region that has large rural and depopulated regions. In this sense, it is worth noting that teacher training in digital competence in Spain is more than double that in other countries, such as those in Latin America (Martín-Párraga et al., 2023).

**Figure 13:**
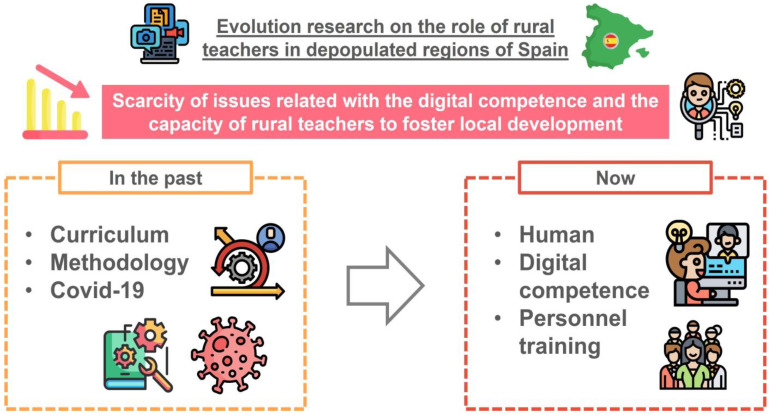
Evolution of research on the role of teachers in rural areas of Spain.

According to the second research objective (RQ2), which is to analyze the main trend lines in terms of research on teachers’ digital competence in depopulated rural areas, it can be stated that a change in trends exists in research related to the digital competence of rural teachers in Spain (Figure 13), which corroborates previous studies developed in other countries (Göltl et al., 2024; Mouraz & Nobre, 2024). The scientific community has left behind research in this area that is focused on curricular and methodological aspects, as well as everything related to the COVID-19 pandemic (Sá et al., 2021; Sá & Serpa, 2020), instead giving way to research focusing on human aspects, the digital competence of teachers, e-learning, and personalized education.

## 5. Limitations of the Study

Among the limitations of this study focused on the digital competence of rural teachers is the fact that access to information sources has been restricted mainly to a single database (Scopus). This could have limited the scope of the analysis, although efforts have been made to ensure that the information selected is representative and of high quality. Furthermore, the available bibliography on the subject in rural contexts is still scarce, which underlines the novelty and relevance of this work, rather than an inherent weakness. Likewise, the geographical limitation of the populations studied is, on the one hand, a necessity for the objectives of the study and, on the other, an obvious limitation placed upon its results. We propose, as a future project, carrying out a comparative analysis of different geographic regions. Likewise, the discussion of the results could be strengthened if this bibliometric review were accompanied by a systematic review and even a field study that would allow a more detailed explanation of the results obtained here. This we propose as a line of future research. The results obtained also offer new possibilities for future research that can expand the theoretical and practical framework developed in this study, consolidating knowledge on digital competence for teachers in rural environments.

## 6. Directions for Future Investigations

Given the importance of digital competence in rural teachers in depopulated areas of Spain, future research could focus on analyzing the self-assessment of the digital competence of rural teachers in Spain over a longer time frame, as well as assessing how new educational policies are impacting this evolution. It is also crucial to conduct longitudinal studies to observe how in-service training programs and professional development policies influence the improvement of teachers’ digital competences over time. In addition, it would be beneficial to conduct comparative research on the digital competences of teachers in rural and urban areas in order to identify gaps and specific opportunities for intervention. Another promising area is the exploration of the impact of emerging technologies, such as artificial intelligence and augmented reality, on improving the digital competence of rural teachers. Finally, qualitative studies are suggested to delve deeper into the barriers and facilitators for the development of digital competences among rural teachers, including socio-economic, cultural, technological, and infrastructural factors (Sánchez et al., 2024).

## 7. Conclusions

Rural teachers, with the right support, have the potential to be significant agents of change, promoting local development initiatives that can range from community entrepreneurship projects to the integration of sustainable technologies into everyday life. Rural teachers need to acquire high digital competence to effectively integrate technology into their pedagogical practices, thus enriching the learning experience of students and encouraging their participation. Digital competence contributes to reducing the educational gap between urban and rural areas, fostering educational equity and inclusion. This conclusion helps to answer RQ1, identifying a poor level of digital competence in teachers in depopulated rural regions of Spain.

The results reveal growing but still insufficient scientific attention to the subject, highlighting the need for rural teachers to acquire digital competencies to adapt to current educational demands. Few researchers are researching the subject and influencing others with their research. The scientific community has left behind research related to the digital competence of rural teachers in Spain that is focused on curricular and methodological aspects, as well as everything related to the COVID-19 pandemic, instead giving way to research on the subject that is focused on human aspects, the digital competence of teachers, e-learning, and personalized education. This conclusion helps to answer RQ2, concerning the main trend lines in terms of research on teachers’ digital competence in depopulated regions of Spain.

It is crucial to increase research in this area in order to design and implement educational approaches that not only improve the quality of education in rural areas but also strengthen the social and economic fabric of these regions. It is essential to have a solid research base that identifies best practices and adapts them to the specific contexts of each community. Promoting rigorous and systematic studies, such as the current one, on this topic will not only make the transformative role of rural teachers visible; it will also ensure that education policies and training programs are developed that effectively empower them to contribute to the sustainable development of their localities.

Castilla y León, a region of Spain with a larger surface area than other European Union member states such as Portugal or Belgium and with an unbalanced population distribution, can serve as a reference to comprehensively address the problem of rural depopulation in Europe. The European Union needs to implement comprehensive and sustainable policies that provide training opportunities and ongoing support to ensure that teachers in rural contexts can acquire or strengthen their digital competence and thus improve the quality of education in these areas. Collaboration between governments, institutions, and local communities is crucial to reverse depopulation trends and ensure a sustainable and prosperous future for Europe’s rural areas.

## Figures and Tables

**Table 1:** Bibliometric database search string.

Scopus Search String
((“digital” OR “digit*”) AND (“compet*”) AND (“education”) AND (“teacher” OR “professor” OR “educator”) AND (“spain” OR “empty spain” OR “rural”))

**Table 2:** Main information from the bibliometric analysis.

**Main Information About Data**	
Timespan	2007:2024
Sources (Journals, Books, etc.)	94
Documents	173
Annual Growth Rate %	11.64
Document Average Age	3.12
Average citations per doc	16.31
**Document contents**	
Keywords Plus (ID)	429
Author’s Keywords (DE)	520
**Authors**	
Authors	504
Authors of single-authored docs	6
**Authors collaboration**	
Single-authored docs	6
Co-Authors per Doc	3.45
International co-authorships %	15.61
**Document types**	
Article	169

**Table 3:** Top five authors’ local impact (2007–2024). TC (total citations), NP (number of publications), and PY (publication year start).

R	Author	h_index	TC	NP	PY_start
1	Cabero-Almenara, J.	9	344	13	2020
2	Palacios, A.	9	399	14	2020
3	Guillén-Gámez, F.D.	8	256	13	2020
4	Barroso-Osuna, J.	4	153	5	2020
5	Mayorga-Fernández, M.J.	4	62	4	2020

**Table 4:** Top five most relevant countries (2007–2024).

R	Country	Articles	SCP	MCP	Freq	MCP-Ratio
1	Spain	96	88	8	0.555	0.083
2	Peru	46	38	8	0.266	0.174
3	China	3	1	2	0.017	0.667
4	Portugal	3	2	1	0.017	0.333
5	Indonesia	2	2	0	0.012	0

**Table 5:** Top five most relevant affiliations (2007–2024).

R	Affiliation	Country	Articles
1	University of Seville	Spain	41
2	University of Granada		33
3	University of Alicante		22
4	University of Burgos		17
5	University of Salamanca		12

**Table 6:** Most relevant countries (2007–2024): TC (total citations) and average article citations.

R	Country	TC	Average Article Citations
1	Spain	1774	18.5
2	United Kingdom	104	52
3	USA	81	40.5
4	Norway	51	25.5
5	Sweden	30	30
6	China	25	8.3
7	Australia	19	19

**Table 7:** Most globally cited documents (2007–2024). TC (total citations), TC per year, normalized TC.

R	Affiliation	Reference	Total Citations	TC per year	Normalized TC
1	Fernández-Cruz, F.J. (2016)	(Fernández-Cruz & Fernández, 2016)	214	23.78	12.54
2	Azorín, C. (2020)	(Azorín, 2020)	157	31.40	9.46
3	Tejedor, S. (2020)	(Tejedor et al., 2020)	121	24.20	6.89
4	Garzón, E. (2020)	(Garzón et al., 2020)	121	24.20	10.08
5	Portillo, J. (2020)	(Portillo et al., 2020)	118	23.60	8.84

## Data Availability

Research data [173 articles selected] are included as supplementary material.

## Supplementary Materials

The following supporting information can be downloaded at: https://www.mdpi.com/article/10.3390/ejihpe15010005/s1.
