# Supplementary material for: Digital Competence of Rural Teachers in Depopulated Regions of Spain: A Bibliometric Review
Source: Eur J Investig Health Psychol Educ. 2025 Jan 7;15(1):5. doi: 10.3390/ejihpe15010005 (PMC11765420; doi:10.3390/ejihpe15010005)
Supplement: Supplementary file 1 [file ejihpe-15-00005-s001.zip › ejihpe-3240291-supplementary.pdf]

Authors	Title
Guillén-Gámez F.D.; Colomo-Magaña E.; Ruiz-Palmero J.; Tomczyk Ł.	The digital competence of the rural teacher of primary education in the mentoring process: a study by teaching speciality and gender
Malmierca M.J.R.; Del Carmen Fernandez Morante M.; Lopez B.C.; Leon F.M.	Cloud Computing and Open SourceSoftware for European Rural Schools
Gil-quintana J.; de León E.V.; Osuna-acedo S.; Marta-lazo C.	Nano-Influencers Edutubers: Perspective of Centennial Generation Families in Spain
Núñez-Canal M.; de Obesso M.D.L.M.; Pérez-Rivero C.A.	New challenges in higher education: A study of the digital competence of educators in Covid times
Martín-Párraga L.; Llorente-Cejudo C.; Barroso-Osuna J.	Self-Perception of Digital Competence in University Lecturers: A Comparative Study between Universities in Spain and Peru According to the DigCompEdu Model
Brazal I.K.; Martinez Monje P.M.; Urrutxi L.D.	Teachers' digital competence and inclusive education at school: An analysis of teacher attitudes
Fernández-Cerero J.; Montenegro-Rueda M.	Digital Competence and Disability: A Qualitative Approach from the Perspective of University Teachers in Andalusia (Spain)
Molina-Torres M.-P.	Flipped learning as a teaching method in the bilingual university classroom
Giacobone G.A.; Pollini A.; Urquiza D.; Chouta K.	Participatory design methods for sustainable interaction design: co-designing digital experiences for sustainability education
Campillo Ferrer J.M.; Miralles Martínez P.	Impact of the flipped classroom model on democratic education of student teachers in Spain
Gabarda Méndez V.; Marín-Suelves D.; Vidal-Esteve M.I.; Ramón-Llin J.	Digital Competence of Training Teachers: Results of a Teaching Innovation Project
Castañeda L.; Villar-Onrubia D.	Beyond functionality: Building critical digital teaching competence among future primary education teachers
Skevi O.; Ortega-Martín J.L.; González-Gijón G.	Use of ICTs and the Digital Competences of Foreign Language Teachers before and during the State of Alarm
Palacios-Rodríguez A.; Guillén-Gámez F.D.; Cabero-Almenara J.; Gutiérrez-Castillo J.J.	Teacher Digital Competence in the education levels of Compulsory Education according to DigCompEdu: The impact of demographic predictors on its development
García-Delgado M.Á.; Rodríguez-Cano S.; Delgado-Benito V.; Lozano-Álvarez M.	Emerging Technologies and Their Link to Digital Competence in Teaching
Paños-Castro J.; Arruti A.; Korres O.	COVID and ICT in Primary Education: Challenges Faced by Teachers in the Basque Country
Fernández-Otoya F.; Cabero-Almenara J.; Pérez-Postigo G.; Bravo J.; Alcázar-Holguin M.A.; Vilca-Rodríguez M.	Digital and Information Literacy in Basic-Education Teachers: A Systematic Literature Review
García-Vandewalle García J.M.; García-Carmona M.; Trujillo Torres J.M.; Moya Fernández P.	Analysis of digital competence of educators (DigCompEdu) in teacher trainees: the context of Melilla, Spain

Chabert, A.	Account of a Foretold Death: Analysing the Response to the Pandemic in the Schools of Castellón (Spain)
Andaluz-Delgado S.; Ordoñez-Olmedo E.; Gutiérrez-Martín N.	Assessment of Digital Teaching Competence in Non-University Education
Syahid A.A.; Herry Hernawan A.; Dewi L.	SMART for the Improvement of Primary School Teachers' Digital Competence in the 21st Century: An Action Research Study
Monteiro A.; Torres A.C.; Clavero S.B.	Digital technologies and school gardens: Possibilities for transformative pedagogies and sustainable development
Boté-Vericad J.-J.; Palacios-Rodríguez A.; Gorchs-Molist M.; Cejudo-Llorente C.	Comparison of the teaching of digital competences between health science faculties in Andalusia and Catalonia
Bachynska N.; Novalska T.; Kasian V.; Maranchak M.; Maranchak N.; Boichuk N.	Peculiarities of Distance Learning Organization in the Professional Training of Information, Librarianship, and Archives (European Experience)
Hu Y.; Nie J.; Gu X.	From Equity of Opportunity to Equity of Development: A Comparative Analysis of Large-Scale Online Education in Urban and Rural K-12 Schools in China during COVID-19
López-Goñi J.J.; Haro B.; Peñalva-Vélez A.; Vega-Osés M.A.	Violent behavior in primary education: Evolution from 9 to 12 years old as a function of gender
Chaves-Yuste B.; de-la Peña C.	Podcasts´ effects on the EFL classroom: a socially relevant intervention
Lozano A.; Blanco Fontao C.	Is the Education System Prepared for the Irruption of Artificial Intelligence? A Study on the Perceptions of Students of Primary Education Degree from a Dual Perspective: Current Pupils and Future Teachers
Fernández-Batanero J.M.; Cabero-Almenara J.; Román-Graván P.; Palacios-Rodríguez A.	Knowledge of university teachers on the use of digital resources to assist people with disabilities. The case of Spain
Fraga-Varela F.; Alonso-Ferreiro A.	Digital Competence in Primary Education and the Limits of 1:1 Computing
Marín-Suelves D.; Méndez V.G.; Monzonís N.C.	Educación Musical y tecnología: tendencias en investigación
Palau R.; Santiago R.; Fretes G.; Mogas J.; Cebrián G.	The Vision of Spanish Schools in Post-Pandemic Times
Martínez-Pérez A.; Lezcano-Barbero F.; Casado-Muñoz R.; Zabaleta-González R.	ICT training in Spanish non-formal education: a revolution in the making
Olmo M.M.; Alba B.G.	Formación en competencia digital del profesorado de educación primaria e infantil en España. Una revisión bibliométrica de la literatura
Ciriza-Mendívil C.D.; Lacambra A.M.; Hernández de la Cruz J.M.	Technological Pedagogical Content Knowledge: Implementation of a Didactic Proposal for Preservice History Teachers
García-Delgado M.Á.; Rodríguez-Cano S.; Delgado-Benito V.; Di Giusto-Valle C.	Digital Teaching Competence among Teachers of Different Educational Stages in Spain
Soekamto H.; Nikolaeva I.; Abbood A.A.A.; Grachev D.; Kosov M.;	Professional Development of Rural Teachers Based on Digital Literacy

Yumashev A.; Kostyrin E.; Lazareva N.; Kvitkovskaja A.; Nikitina N.	
Guillén-Gámez F.D.; Linde-Valenzuela T.; Ramos M.; Mayorga-Fernandez M.J.	Identifying predictors of digital competence of educators and their impact on online guidance
Peñalva S.; Marta-Lazo C.	Analysis of the Level of Critical Competence of Teachers Participating in the Distance and Face-To-Face Education Programme of the European Project "Media in Action", Involving Spain, Italy, Malta, and the United Kingdom
Villar L.B.E.; Herrero L.L.; Álvarez-López G.	UNESCO Strategy and Digital Policies for Teacher Training: The Deconstruction of Innovation in Spain
Salas-Pilco S.Z.; Xiao K.; Hu X.	Correction to: Artificial Intelligence and Learning Analytics in Teacher Education: A Systematic Review
Gallardo-Echenique E.; Tomás-Rojas A.; Bossio J.; Freundt-Thurne Ú.	Evidence of validity and reliability of DigCompEdu CheckIn among professors at a Peruvian private university
Llorente-Cejudo C.; Barragán-Sánchez R.; Puig-Gutiérrez M.; Romero-Tena R.	Social inclusion as a perspective for the validation of the ""DigCompEdu Check-In"" questionnaire for teaching digital competence
Vázquez-Cano E.; Sáez-López J.M.; Grimaldo-Santamaría R.-Ó.; Quicios-García M.D.P.	Influence of Age, Gender and Years of Experience on Teachers in Promoting Strategies for Digital Sustainability and Data Protection
Domene-Martos S.; Rodríguez-Gallego M.; Caldevilla-Domínguez D.; Barrientos-Báez A.	The use of digital portfolio in higher education before and during the COVID-19 pandemic
Milton J.; Giæver T.H.; Mifsud L.; Gassó H.H.	Awareness and knowledge of cyberethics: A study of pre-service teachers in Malta, Norway, and Spain
Méndez D.; Méndez M.; Anguita J.M.	Digital Teaching Competence in Teacher Training as an Element to Attain SDG 4 of the 2030 Agenda
Camilleri P.; Engen B.K.; Hatlevik O.E.; Rubio J.C.C.; Gassó H.H.	Student teachers and their attitudes towards ICT: Lessons learned from three different countries
Binaoui A.; Moubtassime M.; Belfakir L.	The Effectiveness and Impact of Teaching Coding through Scratch on Moroccan Pupils´ Competencies
Martínez-Pérez S.; Cabero-Almenara J.; Barroso-Osuna J.; Palacios-Rodríguez A.	T-MOOC for Initial Teacher Training in Digital Competences: Technology and Educational Innovation
Guillén-Gámez F.D.; Colomo-Magaña E.; Cívico-Ariza A.; Linde-Valenzuela T.	Which is the Digital Competence of Each Member of Educational Community to Use the Computer? Which Predictors Have a Greater Influence?
Gallardo-Montes C.P.; Caurcel Cara M.J.; Rodríguez Fuentes A.; Capperucci D.	Opinions, training and requirements regarding ICT of educators in Florence and Granada for students with functional diversity
Koshkinbayeva N.; Shagataeva Z.; Utepova A.; Taukebayeva K.; Kurmantayeva S.	Exploring pre-service visual art teachers´ competitiveness through porter`s five forces model
Guillén-Gámez F.D.; Mayorga-Fernández M.J.; Contreras-Rosado J.A.	Validez y fiabilidad de un instrumento para evaluar la competencia digital del profesorado en relación a la acción tutorial online en las etapas de educación infantil y educación primaria

Yin Y.; Fang Q.; Li Y.; Cheng H.	A study on health literacy promotion of physical education teachers in rural areas in the context of digital health
Gisbert Caudeli V.; Alayón R.F.; Calderón-Garrido D.; Acero J.M.A.	Teacher involvement in the use of digital tools in conservatory, municipal music school and university classrooms
Montenegro-Rueda M.; Fernández-Cerero J.	Digital Competence of Special Education Teachers: An Analysis from the Voices of Members of School Management Teams
Mateos-Moreno D.; Bravo-Fuentes P.	The 'professional digital competence': Exploring the perspective of primary music teachers in Spain
Puertas-Aguilar M.-Á.; Sipols A.E.G.; de Lázaro-Torres M.-L.	Web GIS to Learn Geopolitics in Secondary Education: A case study from Spain
Guillén-Gámez F.D.; Gómez-García M.; Ruiz-Palmero J.	Competencia digital en labores de Investigación: predictores que influyen en función del tipo de universidad y sexo del profesorado
Campillo-Ferrer J.-M.; Miralles-Martínez P.	Impact of an inquiry-oriented proposal for promoting technology-enhanced learning in a post-pandemic context
Marzo-Navarro M.; Berné-Manero C.	Analysing cross-cutting competencies learning in an online entrepreneurship context
Rubio-Gragera M.; Cabero-Almenara J.; Llorente-Cejudo C.	Study of teachers' digital competence in Official Language Schools in Andalusia (Spain) and its relationships with gender and age variables
Cabero-Almenara J.; Guillén-Gámez F.D.; Ruiz-Palmero J.	Teachers' digital competence to assist students with functional diversity: Identification of factors through logistic regression methods
Valverde-Berrocoso J.; Rivas-Flores J.I.; Anguita-Martínez R.; Montes-Rodríguez R.	Pedagogical change and innovation culture in secondary education: a Delphi study
Martínez-Pérez A.; Lezcano-Barbero F.; Zabaleta-González R.; Casado-Muñoz R.	Usage of ICT among Social Educators—An Analysis of Current Practice in Spain
Guillén-Gámez F.D.; Mayorga-Fernández M.J.	Measuring Rural Teachers' Digital Competence to Communicate with the Educational Community
Rubio-Gragera M.; Cabero-Almenara J.; Palacios-Rodríguez A.	Digital Innovation in Language Teaching: Analysis of the Digital Competence of Teachers according to the DigCompEdu Framework
Bahri A.; Muharni A.; Jamaluddin A.B.; Hidayat W.; Arifin A.N.,	Smart Teaching Based on Lesson Study Promoting Stundent's Digital Literacy in The Rural Area
Guillén-Gámez F.D.; Cabero-Almenara J.; Llorente-Cejudo C.; Palacios-Rodríguez A.	Differential Analysis of the Years of Experience of Higher Education Teachers, their Digital Competence and use of Digital Resources: Comparative Research Methods
Cerero J.F.; Fernández Batanero J.M.; Almenara J.C.	Digital teaching competencies and disability. Validation of a questionnaire design using the K coefficient to select experts
Poveda-Brotons R.; Izquierdo A.; Perez-Soto N.; Pozo-Rico T.; Castejón J.-L.; Gilar-Corbi R.	Building paths to success: a multilevel analysis of the effects of an emotional intelligence development program on the academic achievement of future teachers

Buils S.; Esteve-Mon F.M.; Sánchez-Tarazaga L.; Arroyo-Ainsa P.	Analysis of the Digital Perspective in the Frameworks of Teaching Competencies in Higher Education in Spain
Urrea-Solano M.; Hernández-Amorós M.J.; Merma-Molina G.; Baena-Morales S.	The learning of e-sustainability competences: A comparative study between future early childhood and primary school teachers
Hurtado-Mazeyra A.; Núñez-Pacheco R.; Barreda-Parra A.; Guillén-Chávez E.-P.; Turpo-Gebera O.	Digital competencies of Peruvian teachers in basic education
Borzova A.Yu.; Volosyuk O.V.; Nikolashvili N.D.	Spanish Humanitarian Policy in Latin America: Peculiarities and Priorities
Peters M.; Elasri-Ejjaberi A.; Martínez-Argüelles M.-J.; Fàbregues S.	Teacher digital competence development in higher education: Overview of systematic reviews
Caballero M.L.B.; Gil-Mediavilla M.	Digital storytelling in Spanish language and literature in a primary education university degree: a female perspective
Sáez-López J.-M.; Grimaldo-Santamaría R.-Ó.; Quicios-García M.-P.; Vázquez-Cano E.	Teaching the Use of Gamification in Elementary School: A Case in Spanish Formal Education
Rodríguez N.C.; Lorenzo-Rial M.-A.; Rodríguez U.P.	Digital competence of teachers in terms of content creation: self-perception of teachers in educationalscientific training in Galicia (Spain)
De Juana-Espinosa S.A.; Brotons M.; Sabater V.; Stankevičiūtė Ž.	An analysis of best practices to enhance higher education teaching staff digital and multimedia skills
Omarov B.; Karkulova A.; Ukubassova G.; Abzhan Z.	Innovative approaches in the management of an educational institution
Guerrero Elecalde R.; Contreras García J.; Bonilla Martos A.L.; Serrano Arnáez B.	Digital and Social-Civic Skills in Future Primary Education Teachers: A Study from the Didactics of Social Sciences for the Improvement of Teacher Training in Competences
Alonso-García S.; Victoria-Maldonado J.J.; García-Sempere P.J.; Lara-Lara F.	Student evaluation of teacher digitals skills at Granada University
Martín-Gutiérrez A.; Díaz-Noguera M.D.; Hervás-Gómez C.; Morales-Pérez G.L.	Models of Future Teachers' Adaptation to New Post-Pandemic Digital Educational Scenarios
Gutiérrez-González S.; Coello-Torres C.E.; Cuenca-Romero L.A.; Carpintero V.C.; Bravo A.R.	Incorporating Collaborative Online International Learning (COIL) into Common Practices for Architects and Building Engineers
Zhao, W.	A study of the impact of the new digital divide on the ICT competences of rural and urban secondary school teachers in China
Castillo-Martínez I.M.; Cerros Regalado C.P.; Glasserman-Morales L.D.; Ramírez-Montoya M.S.	Academic literacy among the university students in Mexico and Spain: A holistic perspective
González-Fernández R.; Ruiz-Cabezas A.; Domínguez M.C.M.; Subía-Álava A.B.; Salazar J.L.D.	Teachers´ teaching and professional competences assessment

Dias-Trindade S.; Moreira J.A.; García Huertas J.G.; Garrido Pintado P.; Mas Miguel A.	Teachers´ digital competences in higher education in Portugal and Spain
Samane-Cutipa V.A.; Quispe-Quispe A.M.; Talavera-Mendoza F.; Limaymanta C.H.	Digital Gaps Influencing the Online Learning of Rural Students in Secondary Education: A Systematic Review
Romero Calle D.; Oruna Rodríguez A.M.; Sánchez Ortega J.A.	Digital teaching and learning: Current challenges in Latin America
Fernández-Morante C.; López B.C.; Casal-Otero L.; León F.M.	Teachers' Digital Competence. The Case of the University System of Galicia
Rangel-Pérez C.; Gato-Bermúdez M.-J.; Musicco-Nombela D.; Ruiz-Alberdi C.	The massive implementation of ict in universities and its implications for ensuring sdg 4: Challenges and difficulties for professors
Zhao Y.; Zhao M.; Shi F.	Integrating Moral Education and Educational Information Technology: A Strategic Approach to Enhance Rural Teacher Training in Universitie
Gallardo Montes C.D.P.; Rodríguez Fuentes A.; Caurcel Cara M.J.	ICT training for educators of Granada for working with people with autism
Martinez-Abad F.; Torrijos-Fincias P.; Rodríguez-Conde M.J.	The eAssessment of key competences and their relationship with academic performance
Villalba M.T.; Castilla G.; Redondo-Duarte S.	Factors with influence on the adoption of the flipped classroom model in technical and vocational education
Hinojo-Lucena F.-J.; Aznar-Diaz I.; Caceres-Reche M.-P.; Trujillo-Torres J.-M.; Romero-Rodriguez J.-M.	Factors Influencing the Development of Digital Competence in Teachers: Analysis of the Teaching Staff of Permanent Education Centres
Figueras-Maz M.; Grandío-Pérez M.M.; Mateus J.-C.	Students´ perceptions on social media teaching tools in higher education settings
Romero-Tena R.; Barragán-Sánchez R.; Llorente-Cejudo C.; Palacios-Rodríguez A.	The challenge of initial training for early childhood teachers. A cross sectional study of their digital competences
González Fernández N.; Gozálvez Pérez V.; Ramírez García A.	Media competence of non-university teachers. Diagnosis and training proposals
Becker J.D.,	Digital equity in education: A multilevel examination of differences in and relationships between computer access, computer use and state-level technology
Kuusimäki A.-M.; Uusitalo L.; Tirri K.	Predictors of parental contentment with the amount of encouraging digital feedback from teachers in Finnish schools
Sales D.; Cuevas-Cerveró A.; Gómez-Hernández J.-A.	Perspectives on the information and digital competence of social sciences students and faculty before and during lockdown due to covid-19
Vives-Cases C.; Davo-Blanes M.C.; Ferrer-Cascales R.; Sanz-Barbero B.; Albaladejo-Blázquez N.; Sánchez-San Segundo M.; Lillo-Crespo M.; Bowes N.; Neves S.; Mocanu V.; Carausu E.M.; Pyzalski J.; Forjaz M.J.; Chmura-Rutkowska I.; Vieira C.P.; Corradi C.	Lights4Violence: A quasi-experimental educational intervention in six European countries to promote positive relationships among adolescents
Pozo J.-I.; Pérez Echeverría M.-P.; Cabellos B.; Sánchez D.L.	Teaching and Learning in Times of COVID-19: Uses of Digital Technologies During School Lockdowns

Gordillo A.; Barra E.; López-Pernas S.; Quemada J.	Development of teacher digital competence in the area of e-safety through educational video games
Cordon O.; Anaya K.; Gonzalez A.; Pinzon S.	Promoting the use of ICT for education in a traditional university: The case of the virtual learning center of the University of Grandada
Silva J.B.; Silva I.N.; Bilessimo S.	Technological structure for technology integration in the classroom, inspired by the maker culture
Wang J.; Yang Y.; Li H.; van Aalst J.	Continuing to teach in a time of crisis: The Chinese rural educational system's response and student satisfaction and social and cognitive presence
Stenman S.; Pettersson F.	Remote teaching for equal and inclusive education in rural areas? An analysis of teachersâ€™ perspectives on remote teaching
González M.C.; Martín S.C.; Basantes Andrade A.V.	The Self-Perceived Digital Competence of Social Educators in Spain: Influence of Demographic and Professional Variables
González M.C.; Martín S.C.	Social educators: A study of digital competence from a gender differences perspective
Craig B.; Stevens K.	Learning without limits: The promise of high-speed learning networks for rural and inner-city communities
Portillo J.; Garay U.; Tejada E.; Bilbao N.	Self-perception of the digital competence of educators during the covid-19 pandemic: A cross-analysis of different educational stages
Molise H.; Dube B.	Emergency online teaching in economic and management sciences necessitated by the covid-19 pandemic: The need for healthy relations in a rural schooling context
Suárez-Guerrero C.; Lloret-Catalá C.; Mengual-Andrés S.	Teachers' perceptions of the digital transformation of the classroom through the use of tablets: A study in Spain
Tejedor S.; Cervi L.; Pérez-Escoda A.; Jumbo F.T.	Digital literacy and higher education during COVID-19 lockdown: Spain, Italy, and Ecuador
Baena-Morales S.; Martinez-Roig R.; Hernádez-Amorós M.J.	Sustainability and educational technology: A description of the teaching self-concep
Cabezas-González M.; Casillas-Martín S.; García-Peñalvo F.J.	The digital competence of pre-service educators: The influence of personal variables
Gallego-Arrufat M.-J.; Torres-Hernández N.; Pessoa T.	Competence of future teachers in the digital security area
Pérez-Calderón E.; Prieto-Ballester J.-M.; Miguel-Barrado V.	Analysis of digital competence for spanish teachers at pre-university educational key stages during COVID-19
Pruet P.; Ang C.S.; Farzin D.	Understanding tablet computer usage among primary school students in underdeveloped areas: Students' technology experience, learning styles and attitudes
Casillas-Martín S.; Cabezas-González M.; Muñoz-Repiso A.G.-V.	Digicraft: A pedagogical innovative proposal for the development of the digital competence in vulnerable children
Artacho E.G.; Martínez T.S.; Ortega Martín J.L.; Marín Marín J.A.; García G.G.	Teacher training in lifelong learning-the importance of digital competence in the encouragement of teaching innovation

Guillén-Gámez F.D.; Mayorga-Fernández M.J.; Contreras-Rosado J.A.	Incidence of gender in the digital competence of higher education teachers in research work: Analysis with descriptive and comparative methods
Cabero-Almenara J.; Guillén-Gámez F.D.; Ruiz-Palmero J.; Palacios-Rodríguez A.	Classification models in the digital competence of higher education teachers based on the DigCompEdu framework: Logistic regression and segment tree
Romero-García C.; Buzón-García O.; de Paz-Lugo P.	Improving future teachers' digital competence using active methodologies
Colás-Bravo P.; Conde-Jiménez J.; Reyes-de-Cózar S.	The development of the digital teaching competence from a sociocultural approach
Cabero-Almenara J.; Gutiérrez-Castillo J.-J.; Palacios-Rodríguez A.; Barroso-Osuna J.	Development of the teacher digital competence validation of DigCompEdu check-in questionnaire in the University context of Andalusia (Spain)
López Belmonte J.; Pozo Sánchez S.; Fuentes Cabrera A.	Techno-pedagogical resources to support teaching: Augmented reality as a dynamic tool for the substitute teacher
Stotz S.A.; Brega A.G.; Gonzales K.; Hebert L.E.; Moore K.R.	Facilitators and Barriers to Healthy Eating among American Indian and Alaska Native Adults with Type 2 Diabetes: Stakeholder Perspectives
Lamas J.G.; Cuadrado V.A.	Argumentative skills in the design of webquests in environmental education for secondary students
Cabero-Almenara J.; Guillén-Gámez F.D.; Ruiz-Palmero J.; Palacios-Rodríguez A.	Digital competence of higher education professor according to DigCompEdu. Statistical research methods with ANOVA between fields of knowledge in different age ranges
de Aldecoa C.Y.; Okada A.; Palau R.	New learning scenarios for the 21st century related to education, culture and technology
Thomas-MacLean R.; Hamoline R.; Quinlan E.; Ramsden V.R.; Kuzmicz J.	Discussing mentorship: An ongoing study for the development of a mentorship program in Saskatchewan
Jaén M.M.; Basanta C.P.	An update of ADELEX: Developing the lexical competence of Spanish university students through ICT in the ESHE
Moral F.J.R.; Díaz M.F.	Future primary school teachersâ€™ digital competence in teaching science through the use of social media
Dele-Ajayi O.; Strachan R.; Pickard A.J.; Sanderson J.J.	Games for Teaching Mathematics in Nigeria: What Happens to Pupils' Engagement and Traditional Classroom Dynamics?
Moreno D.; Palacios A.; Barreras A.; Pascual V.	An assessment of the impact of teachers' digital competence on the quality of videos developed for the flipped math classroom
Pettersson F.L.M.	Implementing a swedish regionalized medical program supported by digital technologies: Possibilities and challenges from a management perspective
Fernández-Cruz F.-J.; Fernández-Díaz M.-J.	Generation z's teachers and their digital skills
Candel E.C.; Agustín M.P.; de Ory E.G.	ICT and gamification experiences with CLIL methodology as innovative resources for the

	development of competencies in compulsory secondary education
Lim K.; Kim Y.; Kim M.; Jang Y.; Joo M.-H.	The digital divide? Analyzing regional differences of tablet PC use in Korean middle schools for sustainable development
Ortega-Sánchez D.; Gómez-Trigueros I.M.	Massive open online courses in the initial training of social science teachers: Experiences, methodological conceptions, and technological use for sustainable development
Cabero-Almenara J.; Barroso-Osuna J.; Gutiérrez-Castillo J.-J.; Palacios-Rodríguez A.	The teaching digital competence of health sciences teachers. A study at andalusian universities (Spain)
Alarcón R.; del Pilar Jiménez E.; de Vicente-Yagüe M.I.	Development and validation of the DIGIGLO, a tool for assessing the digital competence of educators
McGarr O.; Mifsud L.; Colomer Rubio J.C.	Digital competence in teacher education: comparing national policies in Norway, Ireland and Spain
López-Belmonte J.; Pozo-Sánchez S.; Ávila-Rodríguez M.; Montero-Cáceres C.	Pedagogical projection of teaching digital competition. The case of a cooperative education
Ortega-Sánchez D.; Gómez-Trigueros I.M.	Didactics of historical-cultural heritage QR codes and the TPACK model: An analytic revision of three classroom experiences in Spanish higher education contexts
García-Galera M.-C.; Martínez-Nicolás M.; Del-Hoyo-Hurtado M.	Innovation in journalism educational programmes at university. A systematic review of educational experiences at Spanish universities
Gómez-ruiz M.-L.; Morales-yago F.-J.; de Lázaro-Torres M.-L.	Outdoor education, the enhancement and sustainability of cultural heritage: Medieval madrid
Guillén-Gámez F.D.; Mayorga-Fernández M.J.	Prediction of factors that affect the knowledge and use higher education professors from spain make of ict resources to teach, evaluate and research: A study with research methods in educational technology
Canfarotta D.; Casado-Muñoz R.	Italian and Spanish studentsâ€™ perception on use of technology in classrooms of classics in secondary school
Gómez-García M.; Hossein-Mohand H.; Trujillo-Torres J.M.; Hossein-Mohand H.	The training and use of ICT in teaching perceptions of melilla's (spain) mathematics teachers
Beardsley M.; Albó L.; Aragón P.; Hernández-Leo D.	Emergency education effects on teacher abilities and motivation to use digital technologies
Cabero-Almenara J.; Barroso-Osuna J.; Palacios-Rodríguez A.	Digital competences of educators in Health Sciences: Their relationship with some variables
Sáez-López J.-M.; Feliz-Murias T.; Holgueras-González A.-I.	Interactive videoconferencing in educational settings: A case in primary education
Calderón-Garrido D.; Gustems-Carnicer J.; Carrera X.	Digital technologies in music subjects on primary teacher training degrees in Spain: Teachersâ€™ habits and profiles
Del-Moral-Pérez M.E.; Villalustre-Martínez L.; Neira-Piñeiro M.D.R.	Teachers´ perception about the contribution of collaborative creation of digital storytelling to the communicative and digital competence in primary education schoolchildren

Urbieta A.S.; Peñalver E.A.	Multimodal discourse in digital storytelling: An assessment tool proposal
Prieto-Ballester J.-M.; Revuelta-Domínguez F.-I.; Pedrera-Rodríguez M.-I.	Secondary school teachers self-perception of digital teaching competence in spain following covid-19 confinement
Seymour-Walsh A.E.; Weber A.; Bell A.	Pedagogical foundations to online lectures in heaith porfessions education
Sáez-López J.-M.; Rodriguez-Torres J.	Reviews of educational policy regarding one laptop per child: Escuela 2.0 program in Castilla-La Mancha, Spain
Azorín C.	Beyond COVID-19 supernova. Is another education coming?
Morueta R.T.; Gómez J.I.A.; Gómez A.H.	B-learning at universities in Andalusia (Spain): From traditional to student-centred learning
Novella-García C.; Cloquell-Lozano A.	The ethical dimension of digital competence in teacher training
Palomino M.C.P.	Information and communication technologies and inclusive teaching: Perceptions and attitudes of future early childhood and primary education teachers
Gudmundsdottir G.B.; Gassó H.H.; Rubio J.C.C.; Hatlevik O.E.	Student teachers' responsible use of ICT: Examining two samples in Spain and Norway
Garzón-Artacho E.; Sola-Martínez T.; Romero-Rodríguez J.-M.; Gómez-García G.	Teachers' perceptions of digital competence at the lifelong learning stage
Rojo-Ramos J.; Carlos-Vivas J.; Manzano-Redondo F.; Fernández-Sánchez M.R.; Rodilla-Rojo J.; García-Gordillo M.Á.; Adsuar J.C.	Study of the digital teaching competence of physical education teachers in primary schools in one region of Spain
Del-Moral M.E.; Villalustre L.; Neira-Piñeiro R.	Instruments to measure competences in digital storytelling design at preschool and primary education: The CINEMA project
